# Recent Advances in Bio-Based Adhesives and Formaldehyde-Free Technologies for Wood-Based Panel Manufacturing

**DOI:** 10.1007/s40725-024-00227-3

**Published:** 2024-07-30

**Authors:** Ingrid Calvez, Rosilei Garcia, Ahmed Koubaa, Véronic Landry, Alain Cloutier

**Affiliations:** 1 Department of Wood and Forest Sciences, Renewable Materials Research Centre (CRMR), Faculty of Forestry, Geography, and Geomatics, Université Laval, 2425 Rue de La Terrasse, Quebec, QC G1V 0A6 Canada; 2 Forest Research Institute, Université du Québec en Abitibi-Témiscamingue (UQAT), 445 Boulevard de l’Université, Rouyn-Noranda, QC J9X 5E4 Canada

**Keywords:** Bio-based adhesives, Wood panels, Coproducts, Bio-waste, Binderless panels, Bio-scavengers

## Abstract

**Purpose of Review:**

Conventional formaldehyde-based adhesives for wood-based composite panels are subject to significant concerns due to their formaldehyde emissions. Over the past decade, the wood adhesive industry has undergone a considerable transformation that is characterized by a major push in bio-adhesive development. Various bio-based materials have been explored to create alternatives to conventional formaldehyde-based adhesives. Moreover, growing interest in circularity has led to increasingly exploiting industrial coproducts and by-products to find innovative solutions.

**Recent Findings:**

Industrial production generates many coproducts that can serve as renewable resources to produce eco-friendly materials. These coproducts offer alternative supply sources for material production without encroaching on food production. Many bio-based compounds or coproducts, such as saccharides, proteins, tannins, and lignocellulosic biomass, can also be used to develop bio-based adhesives. As part of ongoing efforts to reduce formaldehyde emissions, new hardeners and crosslinkers are being developed to replace formaldehyde and bio-scavengers. Other alternatives, such as binderless panels, are also emerging.

**Summary:**

This review focuses on sources of bio-based material derived from by-products of various industries, which have many advantages and disadvantages when incorporated into adhesives. Modification methods to enhance their properties and performance in wood-based panels are also discussed. Additionally, alternatives for developing low-emission or formaldehyde-free adhesives are addressed, including hardeners, bio-scavengers, and binderless options. Finally, the environmental impact of bio-based adhesives compared to that of synthetic alternatives is detailed.

## Introduction

Many international organizations have described the health risks associated with formaldehyde emissions (FEs), including the International Agency for Research on Cancer (IARC) and the World Health Organization (WHO). Indeed, IARC classified formaldehyde as a Group 1 carcinogen for humans in 2004 [1]. In 2010, the WHO established an indoor air quality guideline for short- and long-term exposure to formaldehyde of 0.1 mg/m^3^ (0.08 ppm) for all 30-min periods at lifelong exposure [2]. In the United States, the California Air Resources Board (CARB) implemented a measure in 2009 to control airborne toxic substances and limit FEs from composite wood products sold in California. Canada subsequently aligned its regulations with the those of United States to protect human health and the Canadian economy. The *Canadian Environmental Protection Act* established maximum emission levels for formaldehyde from composite wood products, which range from 0.05 to 0.13 ppm depending on the type of product [3]. Conventional adhesives are the primary target of these regulations. These adhesives include phenol–formaldehyde (PF), urea–formaldehyde (UF), melamine–formaldehyde (MF), and polymeric diphenylmethane diisocyanate (pMDI) resins. While these adhesives are cost-effective and perform well in terms of bonding ability, mechanical properties, thermal stability, and water resistance, stricter regulations are driving industries and researchers to develop new bio-based adhesives to meet the growing demand for healthier and more environmentally friendly products. There has been a substantial increase in the number of scientific papers investigating bio-based adhesives for wood-based composite panels (WBCPs) since 1990. There were approximately 60 papers on the topic in 2000, and more than 500 in 2022. This surge can be primarily attributed to the implementation of new FE regulations worldwide and growing interest in using bio-based and renewable products as alternatives to petroleum-derived products. Various sources of plant- and animal-based raw materials are being explored to produce low-emission or formaldehyde-free bio-based adhesives. These materials include lignocellulosic compounds, tannins, plant- or animal-based proteins, saccharides, and starch. This review summarizes recent advances in bio-based adhesives for WBCP manufacturing, and the environmental impacts and current challenges associated with such adhesives.

## Overview of the WBCP/Adhesive Market

Many types of WBCPs exist for a variety of applications. This section reports on particleboard (PB), medium-density fiberboard (MDF), oriented strand board (OSB), plywood, and their conventional adhesives. The main adhesive systems used in the particleboard and MDF industry are UF, MF, melamine-urea–formaldehyde (MUF), PF, and pMDI. UF adhesives are the thermosetting resins most commonly used to produce plywood and other interior-grade WBCPs (PB and MDF), and represent nearly 85% of all aminic resins manufactured globally, with an annual volume of around 11 million tons. Global production of UF-bonded MDF exceeds 100 million cubic meters per year. Over 90% of WBCPs produced worldwide are made with UF adhesives [4]. The forest products industry utilizes UF resin extensively, accounting for 61% in particleboard, 27% in MDF, and 5% in hardwood plywood production. Additionally, it serves as a laminating adhesive (7%) for bonding furniture overlays to panels and interior flush doors [5]. The WBCPs used in outdoor applications, like plywood and OSB, are produced with hydrolytically stable resins like PF (plywood, OSB) and pMDI (OSB) [6, 7].

## Recent Developments in Bio-based Adhesives

### Utilization of Renewable Natural Resources

#### Valorization of Industrial Coproducts and Bio-waste

**Fig. 1:**
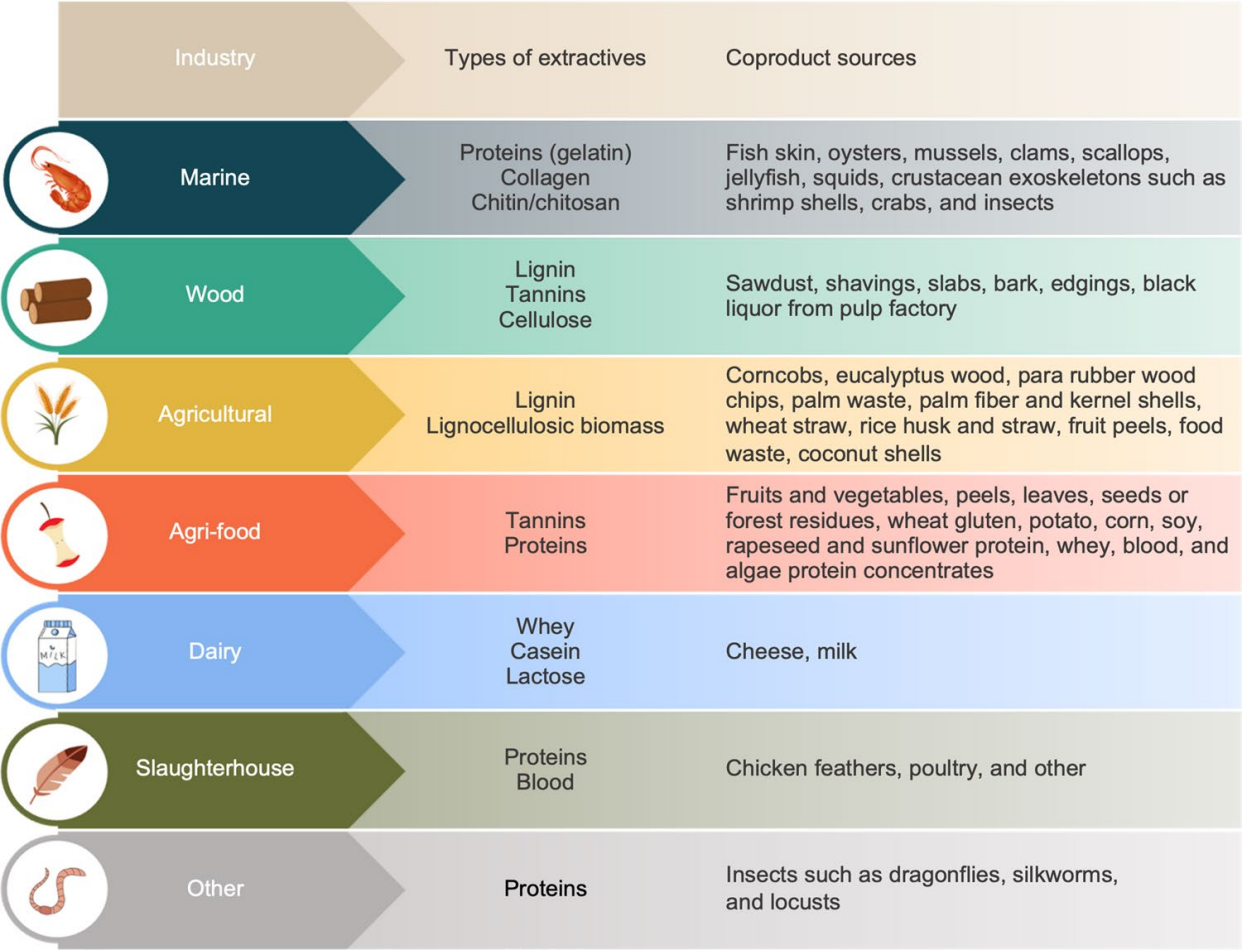
Overview of sources of coproducts extracted from several industries

Several renewable natural materials are at the forefront of new bio-based adhesive developments for WBCPs [8]. Nevertheless, a significant portion of these natural materials is derived from intensive agriculture or unsustainable production practices, or in direct competition with resources intended for human or animal consumption. This section details the process of valorizing industrial coproducts and bio-waste, whose sources are set out in Fig. 1. This process involves diverting these resources from their usual path towards the landfill or incinerator and thus reduces a significant cost burden for the industries that are responsible for their disposal.

–Marine Biowaste

During commercial processing, the seafood industry produces substantial solid discards and by-products, constituting up to 60% proteins on a dry-weight basis [9–12]. These proteins include myofibrillar protein, collagen, enzymes, and soluble nitrogenous compounds [9]. Chitin, the second most abundant polysaccharide in nature, is sourced from crustacean exoskeletons, notably shrimp shell waste [13].

–Wood Residues

Wood compounds, such as lignin, tannins, and cellulose, can be readily extracted from wood processing residues [14–16]. In fact, for every 2.4 m^3^ of lumber produced, sawmills collect around 907 kg of sawdust, shavings, slabs, and edgings. About 75% of this material is wood leftovers, while the remaining 25% is bark. [14]. These wood residues can be used to generate energy; manufacture WBCPs, pulp, and paper; and produce various bio-products, including bio-adhesives for WBCPs [17].

–Agricultural Industry

Agricultural waste poses a considerable challenge, as humans annually generate 150 billion metric tons through intensive farming, harvesting, cultivation, and industrial practices [18–20]. Unfortunately, a substantial portion of this waste is improperly disposed of through random burning or landfilling, leading to environmental pollution. However, efforts towards sustainability are being made, particularly by using agricultural biowaste such as starch, cellulose, and protein to produce natural biopolymers [18].

–Agri-Food Industry

Agri-food by-products, such as discarded fruits and vegetables, seeds, leaves, peels, and forest residues, are potential sources of high-value bioactive molecules such as tannins. Furthermore, the main sources of protein derived from biowaste and by-products from the agri-food industry include wheat, potato, corn, soy, rapeseed, and sunflower [21–23].

–Dairy Coproducts

In the dairy sector, milk processing yields numerous by-products, that could represent potential environmental concerns if not disposed of properly. Among these, the cheese industry stands out for producing the most significant and environmentally impactful by-product: whey. Nevertheless, there has been significant focus in recent decades on the valorization of whey and its by-products, driven by the rising demand for protein-rich products. However, the increased production of whey protein products worldwide means there has also been an increase in the production of by-products from these processes, such as whey permeates, which are mainly composed of lactose [24]. Aside from the usual by-products resulting from standard milk processing, significant quantities of milk are occasionally discarded due to non-compliance with food industry safety standards or surplus production. This discarded milk contains valuable components like casein, whey protein, and lactose, presenting promising prospects as a raw material for various applications [25].

–Slaughterhouse – Blood, Chicken Feathers

Chicken feather waste, a material whose global annual production in the poultry industry represents 65 million tons, is primarily dumped, buried, or burned. However, this biowaste could be a promising source of value-added products [26] such as the protein keratin, which has a unique structure. Its high disulfide bond content means its crosslinking can be broken and re-established [27–29]. Blood is one of the poultry industry’s main by-products, and it is usually underutilized due to high disposal cost. The unsafe and improper disposal of such waste can lead to tremendous environmental pollution and associated health challenges. Many slaughterhouses opt to dispose of poultry blood in landfills, which leads to this protein source being underutilized.

–Other

While insects are considered a dietary staple in certain cultures, they are generally regarded as unwanted pests or an unhealthy food option in many regions due to their potential consumption of toxic plants or as they live in pesticide-treated environments [21]. García et al. investigated the potential of using black soldier fly larvae as a new alternative to protein-based adhesives and a viable substitute for UF resin in particleboard production [30].

#### Biopolymer Extraction: Traditional vs. Green Processes

Considerable resources have been devoted to developing simple, cost-effective approaches to extract biopolymers from waste biomass. Various solvents and extraction techniques, including high-pressure and high-temperature techniques, supercritical fluids, ultrasound- and microwave-assisted extraction, as well as enzymatic treatments, have been suggested to more efficiently retrieve valuable compounds. Traditional organic solvents such as diethyl ether, N,N-dimethylformamide, ethanol, hexane, toluene, and their aqueous solutions are commonly used as extractants. Nevertheless, many solvent-based extraction methods are criticized for being inefficient due to their prolonged extraction/purification times and the fact that they need a substantial volume of solvent per sample, which results in a considerable amount of toxic waste being generated [31]. For example, conventional chitin extraction using outdated chemical processes necessitates large quantities of hazardous chemicals (NaOH and HCl) that generate residues and requires lots of water in each process stage for neutralization and washing [32]. However, a variety of methods have been developed that successfully use environmentally friendly techniques to extract chitin and chitosan from various sources. These methods include ionic liquids, deep eutectic solvents, microbial fermentation, enzyme-assisted extraction, microwave-assisted extraction, ultrasonic-assisted extraction, subcritical water extraction, and electrochemical extraction [32, 33].

#### Technologies for Modifying Natural Compounds and Their Bio-Based Adhesives

A comprehensive summary of the technologies that are employed to modify lignin and proteins is provided in Table 1. Many lignin modification methods, such as demethylation, hydroxymethylation, phenolation, and oxidation, have been reported to effectively improve lignin’s reactivity. For example, demethylation is an effective prospective method whereby phenolic hydroxyl groups are generated under mild reaction conditions through the conversion of aromatic methoxy groups. Consequently, the lignin has more reactive sites, with a higher proportion of catechol moieties, which enhances its reactivity [34]. Furthermore, various protein modification methods exist, including thermal, chemical, enzymatic, and mechanical techniques. Protein modification enhances the water resistance and bonding strength of protein molecules by exposing specific functional groups. Modification occurs by breaking down the primary protein structure, which reduces the molecular size of the protein and results in reactive amino and carboxyl groups being exposed [35, 36].

**Table 1:** Enhancing Lignin and Proteins: Modification Types, Processes, and Applications

Raw material	Type of modification	Process and application	Reference
Lignin	Demethylation	Biotic microorganisms, such as fungi, convert aromatic methoxy groups present in lignin into phenolic hydroxyl groups under mild reaction conditions	[34]
	Methylation	To introduce functional hydroxymethyl groups to lignin	[34]
	Phenolation	The condensation between phenol and lignin generates additional reactive sites and initiates the cleavage of ether bonds	[37]
	Depolymerization	This approach involves hydrolyzing lignin into lower molecular weight fragments, resulting in the cleavage of ether bonds, aryl–alkyl bonds, aryl-aryl bonds, and alkyl-alkyl bonds that connect the phenylpropane units of lignin. The microwave-assisted digestion (MWD) method is used to obtain phenol-enriched hydroxy depolymerized lignin and promote the oxidation and digestion of lignin	[34, 38]
	Oxidation	Electrochemical oxidation. When the oxidant NaIO_4_ is used, aldehyde groups are generated that are capable of reacting with demethylated lignin to increase crosslinking and hardness	[39, 40]
	Steam explosion	This method results in low molecular mass and polydispersity, reduced recondensation, and enhanced crosslinking reactivity in green adhesive formulations	[41]
	Maleic anhydride (MA)	MA has a highly reactive aromatic ring and unsaturated double bonds in its structure. It can react with free and linked formaldehyde and phenol in PF resin. A lignin-based polyacid catalyst grafted with MA has been shown to successfully catalyze polycondensation reactions during UF resin formation	[42, 43]
	Thiol-ene "click" chemistry	This method involves functionalizing lignin with terminal alkyne groups and then facilitating crosslinking with a multifunctional thiol to form a polymeric network	[44]
Proteins	Physical denaturation	By freezing, heating, high pressure, shear radiation, or ultrasonic treatment	[36]
	Chemical denaturation	Hydrolysis leads to the cleavage of peptide bonds, which form the protein's primary structure. It occurs when the carboxyl groups (-COOH) in the protein are neutralized, forming carboxylate anions under alkaline conditions, which generate repulsive forces between the anions. The primary alkali denaturing reagents include NaOH, Ca(OH)_2_, ammonia, and borax	[36, 45]
		Surfactant and denaturant agents – The hydrophobic groups of the surfactant can interact with those of protein molecules, leading to protein unfolding. This exposure of the protein's nonpolar groups to the medium results in a stable structure in water, consequently enhancing wettability Urea (U), sodium dodecyl sulfate (SDS), and sodium hydrogen sulfite (SHS) – The action of urea, SDS, and SHS destroy the protein molecule's hydrogen bond, which increases the surface hydrophobicity	[35, 46]
		Ethanol – Its hydrophobic group can be immersed in the protein molecule, which destroys the molecular structure of the protein molecule, leads to protein denaturation, and exposes the protein molecule's hydrophobic amino acid residues	[47]
	Crosslinking	Crosslinking involves predominantly reactions between the crosslinking agent and polar groups of the protein, including -OH, -NH_2_, -COOH, and -SH, thereby enhancing the average functionality and crosslinking density	[35]
	Grafting	Grafting involves introducing active sites onto the protein molecules through chemical reactions, which can react with active groups or monomers with double bonds	[35]
	Oxidation	Oxidant modification converts carbohydrates into aldehyde groups via oxidation, and these aldehyde groups subsequently undergo crosslinking with the active groups of protein molecules, enhancing the adhesive's bonding performance	[35]
	Enzyme treatment	This process involves modifying the protein's structure or composition by protease hydrolysis or polymerization catalysis under mild conditions. Protease modification employs biological methods to eliminate or incorporate groups into the amino acid or polypeptide chain, thereby modifying its physical or chemical properties	[35, 36]
	Organic–inorganic hybridization	Nanoscale particles are used to enhance the adhesive's bond strength, thermal behaviour, and water resistance due to their unique nanoscale structure, high particle aspect ratio, and formation of inorganic–organic hybrids. Nanoscale montmorillonite, kaolin, attapulgite, halloysite nanotubes, aluminum hydroxide nanoparticles, and metakaolin-based geopolymer have been reported to be promising modifiers to improve the bond properties of soybean-based adhesives	[48–52]

–Carbohydrates: Starch, Chitosan, Sucrose, and Others

Three types of carbohydrates exist, namely monosaccharides (e.g., glucose, fructose, galactose), disaccharides (e.g., lactose, sucrose, maltose), and polysaccharides [53, 54]. Starch is a polysaccharide composed of D-glucose units (glucopyranose), with amylose and amylopectin as its primary biopolymers. The properties of starch-based adhesives hinge on the ratio of amylose to amylopectin. Due to carbohydrate molecules’ polar structure, carbohydrate adhesives typically absorb considerable amounts of water, leading to weakened and poor wet bond strength. Modifying agents are commonly incorporated for two main purposes: to induce gelatinization by breaking down starch before application and to directly alter the starch structure.

Chemical modification by crosslinking, grafting copolymerization, oxidation, esterification, and etherification, among other methods, has been employed to improve the performance of starch-based adhesives. Typically, starch-based adhesives are produced through esterification or transesterification reactions, where the hydroxyl groups of starch are replaced with larger functional groups derived from free fatty acids or their derivatives. Acid hydrolysis of starch leads to a reduction in its molecular weight as it breaks down into amylose and amylopectin [55]. To minimize hydrogen interaction and enhance the mechanical properties of starch-based adhesives, the hydroxyl groups within starch molecules can be oxidized to carbonyl and carboxyl groups. [56]. Graft-modifying starch with hexyl acrylate or glycidyl methacrylate is expected to improve its hydrophobicity [57].

**Fig. 2:**
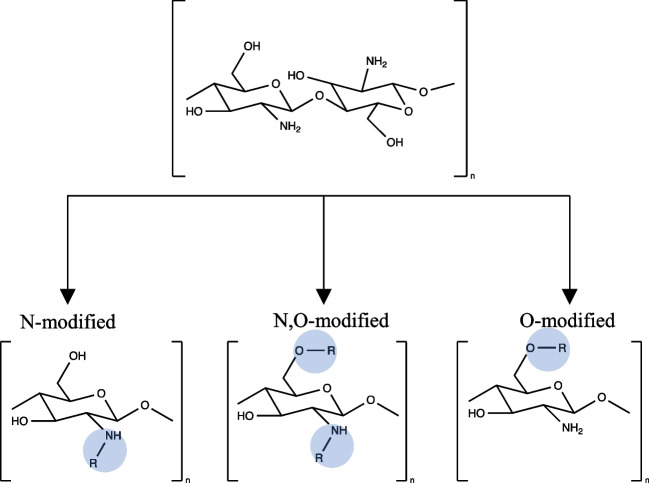
Structure and chemical modification of chitosan by amino groups as well as primary and secondary hydroxyl groups at the C-3 and C-6 positions [60]

Chitosan, consisting of β-(1,4)-linked 2-acetamido-2-deoxy-d-glucopyranose and 2-amino-2-deoxy-d-glucopyranose units, is obtained from chitin via deacetylation using alkaline treatment. A significant drawback of chitosan solutions in the adhesive is their rapid solidification within seconds when mixed with reactive low-molecular-weight aldehydes (such as formaldehyde, glyoxal, and glutaraldehyde) used as hardeners. This characteristic makes it impossible to spread them onto the wood surfaces. [58]. Chitosan, rich in hydroxyl and amino groups, exhibits strong chemical reaction abilities in terms of intramolecular and intermolecular hydrogen bond formation. Chitosan can undergo crosslinking with acids such as citric and boric acid, along with aldehydes like glyoxal and glutaraldehyde, to improve adhesive’s strength and moisture resistance [59]. Furthermore, chitosan can be chemically modified thanks to its amino and primary and secondary hydroxyl active groups, as shown in Fig. 2 [60]. Several chemical modifications can occur, such as Schiff base modification, carboxylation, alkylation, acylation, quaternization, graft modification and crosslinking.

Sucrose-based adhesive crafted by ester linkages with citric acid and sucrose has been shown to exhibit excellent performance on particleboard. Nonetheless, the challenge associated with esterifying hydroxyl and carboxyl groups prompted the utilization of high press temperature (200 °C) and extended press time (10 min) to ensure effective adhesive curing. An alternative method involves thermally degrading sucrose to obtain 5-hydroxymethylfurfural (5-HMF) [61]. Indeed, dehydrating carbohydrates, particularly monosaccharides and oligosaccharides, can yield 5-HMF and other furan compounds under acidic conditions. The sucrose’s glycosidic linkage undergoes cleavage in the presence of acid, such as citric acid, producing fructose and glucose compounds. Subsequently, under hot pressing conditions, these compounds are converted into 5-HMF through dehydration mechanisms. This research underscores the various approaches that can be used to optimize sucrose-based adhesives for different applications and considers both esterification and thermal degradation pathways [62]. As Song et al. studied, sucrose can be oxidized by an oxidant such as sodium permanganate (KMnO_4_), hydrogen peroxide (H_2_O_2_), sodium periodate (NaIO_4_), ammonium persulfate ((NH_4_)_2_S_2_O_8_) or ammonium nitrate (NH_4_NO_3_) to obtain bio-based aldehydes [61]. Song et al. demonstrated that (NH_4_)_2_S_2_O_8_ successfully oxidizes native sucrose and that the hydroxyl groups are transformed into carbonyl or carboxyl groups. In their experiments, the products of oxidized sucrose included formaldehyde, glyoxal, formic acid, ethanedioic acid, and oxaloacetic acid. Then, the oxidized sucrose reacted with hexanediamine by Schiff base reaction and amide reaction [61]. Moreover, Zhang et al. developed wood adhesives derived from biomass that showcased excellent bonding properties and water resistance. They used sodium periodate as an oxidant at a low reaction temperature (50 °C) to treat carbohydrates like glucose, sucrose, and starch. The treatment resulted in the formation of solutions containing biomass-derived aldehydes. The authors then prepared three distinct resins—oxidized glucose-hexamethylenediamine, oxidized sucrose-hexamethylenediamine, and oxidized starch-hexamethylenediamine—through a Schiff base reaction with the solutions and hexamethylenedimine [63].

–Tannins: Bio-Based Adhesive Formulations

Tannins are another renewable material that can serve as bio-based adhesive in WBCP manufacturing. Tannins can be classified into two types: hydrolyzable and condensed tannins. Hydrolyzable tannins find application in the leather industry, whereas condensed tannins are employed in adhesive synthesis due to their unique chemical composition. Derived from the polymerization of flavanol monomers, condensed tannins possess phenolic hydroxyl groups and exhibit high reactivity attributed to the resorcinol and phloroglucinol functionality in their structure. Among condensed tannins, mimosa bark, quebracho heartwood, and pine bark tannins have been subject to extensive research for the development of bio-based adhesives [8, 64].

However, their utilization is limited due to their high viscosity and poor water resistance, attributed to the presence of hydrogen bonds and electrostatic interactions within their structure. Tannin-based resins form a crosslinked structure by autocondensation or crosslinking. Tannin autocondensation reactions occur through the generation of ions along the polymeric chain in an acidic or basic environment and are initiated by opening the flavonoid repeating unit. Subsequently, these structures interact with other tannin chains through ionic polycondensation. Furthermore, incorporating a crosslinker in tannin-based resin has been shown to increase its mechanical strength, such as internal bonding (IB). Innovations in crosslinkers and hardeners are detailed in the next section. A range of crosslinkers, such as hexamethylenetetramine, hexamine, trishydroxymethyl nitromethane, glyoxal, glutaraldehyde, phthaldialdehyde, caprolactam, chitosan, dicyandiamide, citric acid, maleic anhydride, phthalic acid, furfural, and furfuryl alcohol, have been explored as hardeners for tannin-based resins [64–67]. Moreover, hybrid tannin-based adhesives are commonly used in WBCPs such as PB and plywood. Furanic derivatives, such as 5-HMF produced from the conversion of sucrose during hot pressing, have been used as crosslinkers in tannin resin formulations [68, 69]. Another bioresource that is frequently studied with tannins is starch. Tannins have been proven to help address the drawbacks associated with starch, including inadequate water resistance, limited shelf life, high viscosity, and low mechanical properties. Incorporating starch makes it possible to utilize less tannin in resin synthesis [70, 71]. Moreover, using tannins with soy protein and lignin has been investigated in many studies [71–73].

## Low-emission and Formaldehyde-free Adhesives

### Innovations in Hardeners and Crosslinkers

**Fig. 3:**
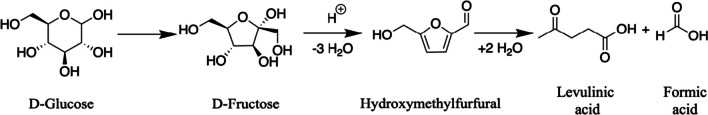
Acid-catalyzed dehydration of monosaccharides to 5-HMF and rehydration to produce levulinic acid and formic acid [80]

Acetaldehyde, propionaldehyde, n-butyraldehyde, glyoxylic acid, glycolaldehyde, and vanillin can be used as crosslinkers instead of formaldehyde in adhesives. Partial substitution of formaldehyde by n-butyraldehyde has been shown to improve the water resistance of the adhesive [74]. The non-volatile aldehyde glyoxal is utilized in adhesives such as protein-glyoxal and lignin-glyoxal resins for WBCPs [75, 76]. Studies have demonstrated that glyoxal is less reactive than formaldehyde. Dimethoxyethanal (DME), an aldehyde derived from glyoxal, is a non-toxic compound employed in the production of WBCPs. DME can effectively be used as a substitute for formaldehyde in melamine and urea resins with a single formyl group. Although DME reacts with melamine and urea in a similar pH range as formaldehyde, it is notably less reactive [77]. Glutaraldehyde is an effective crosslinking agent for proteins thanks to its ability to react with amino groups [6]. Glycolaldehyde (GA), which is derived by cracking glucose, has been tested as a potential substitute for formaldehyde in UF adhesives. However, Sandahl et al. showed that it is not a good substitute for formaldehyde in UF resins. The low bond strength and water solubility urea-GA resins have been found to exhibit, which may be attributable in part to the incomplete polymerization of the resin and the hydrophilic nature of the hydroxyl group in GA, render GA-based resins more hydrophilic than formaldehyde-based adhesives [78]. Low-mass furanic compounds like furfuraldehyde, furfuryl alcohol, and 5-HMF are derived by treating carbohydrates such as polysaccharides, gums, and sugars with acid [79]. Furthermore, the acid-catalyzed dehydration of hexoses, such as D-Glucose or D-Fructose, leads to 5-HMF (see Fig. 3) [80]. However, resins that contain a furanic compound as a substitute for formaldehyde require longer press times and high press temperatures (180 °C for 5-HMF and 130 °C for furfural) to cure because these bio-based aldehydes are less reactive.

### Bio-Scavengers

Adding bio-based additives such as tannins, lignin, starch, wheat flour, and rice husk flour, or other compounds such as starch derivatives, charcoal, pozzolan, and zeolites to resin is an eco-friendly way to decrease FEs from WBCPs [81]. Bark flour from tree species with high polyphenol content (walnut, chestnut, fir, and spruce) also exhibit formaldehyde-scavenging properties. Adding mimosa and birch bark to UF adhesives is an inexpensive solution for valorizing bark on an industrial level as a bio-based formaldehyde scavenger [82, 83]. Moreover, chitosan is a biodegradable and eco-friendly scavenger that reduces FEs. Chitosan powder was found to have greater formaldehyde adsorption capacity than chitosan solution [59]. The reaction between chitosan amino groups and formaldehyde carbonyls results in the reduction of FE [84]. Various nano-adsorbents for formaldehyde, most of which are polysaccharide-based, have been developed in this context. Shalbafan et al. specifically prepared chitosan grafted with 3-[2-(2-aminoethylamino)ethylamino]propyl-trimethoxysilane (AAAPTMS) and chitosan nanoparticles to increase formaldehyde adsorption efficiency. They showed that their MDF panels' overall FEs decreased when 1% chitosan-based adsorbents were added to the UF resin. Furthermore, chitosan's amino reactive sites mean grafting and many other modifications such as alkylation, hydroxy alkylation, carboxy alkylation, sulfation, thiolation, phosphorylation, acylation, and acidic or enzymatic depolymerization can be performed under mild reaction conditions to create new functionalized derivatives [85]. Primary or secondary amine group-containing scavengers, including urea, ammonia, and melamine, are widely employed and cost-effective for reducing FE. Their efficiency stems from their ability to react with free formaldehyde in the resin and hydrolyzed formaldehyde in the panel. Therefore, proteins, which are composed of amino acids, can be used as scavengers and reduce the FEs from WBCPs [86]. Liu et al. developed formaldehyde scavenger microcapsules to prepare ultra-low-FE particleboard. Their results showed that a microencapsulated formaldehyde scavenger prepared by emulsification crosslinking with chitosan as the wall material and urea as the core material can effectively control formaldehyde release over 180 days. Compared to their control panel, their particleboard containing the scavenger microcapsules reduced FEs by 51.4% at 28 days and 25.8% at 180 days. It is worth noting, however, that introducing microcapsules had an adverse impact on the particleboard's physical and mechanical properties [87]. The use of nanocellulose modified with aminosilane (3-aminopropyltriethoxysilane, APTES) as a formaldehyde scavenger in UF resin was studied. Kawalerczyk et al. showed that functionalizing nanocellulose with APTES results in a reduction in FEs [88]. Dukarska et al. investigated adding pine needles as a formaldehyde scavenger to UF resin using the same principle. Since non-modified needles negatively impact the bonding quality of plywood, they modified the pine needles with APTES and hydrothermal treatment. Their study showed that applying hydrothermal treatment and silanization to the pine needles improved the plywood's bonding quality, increased its shear strength, reduced its tendency to delaminate after the aging test, and significantly reduced its FEs [89]. The effectiveness of green tea in reducing FE was also confirmed in a study, where green tea leaves were used as a filler in plywood [90].

### Nanocellulose-reinforced Wood Adhesives

Adding the sustainable and low-cost bio-nanomaterial nanocellulose (NC), either as cellulose nanocrystals (CNCs) or cellulose nanofibers (CNFs), into formaldehyde-based adhesives for WBCPs can increase the mechanical and physical properties of the panels while also reducing their FEs [91]. However, NC's use as adhesive reinforcement is limited because it significantly increases the viscosity of adhesives [92]. Lengowski et al. studied the addition of CNFs at different concentrations to reinforce PF resin for plywood panels. They found that adding CNFs increased the viscosity and decreased the gel time of the adhesives considered, which could interfere with adhesive penetration into the wood. However, in their study, the CNFs improved the adhesives' interaction with the wood and resulted in greater resistance in dry and wet shear tests. They showed that the parallel and perpendicular static bending strengths of their CNF-reinforced treatments were the same as those of their CNF-free control treatment [93]. Furthermore, Kawalerczyk et al. investigated the effect adding NC to MUF adhesives has on the properties of plywood. They demonstrated that adding small amounts of NC improved the panels' bonding quality, mechanical properties, and bending strength, and decreased their FEs [94]. Yildirim et al. investigated how adding NC and boric acid (BA) to UF resin affected the mechanical and physical properties of particleboard. Their results showed that using NC and BA as reinforcements positively affected the mechanical and physical performance of the panels, for example, by increasing IB with the addition of 3% NC and 3% BA [95]. Finally, the benefits that incorporating NC in wood adhesives provides open up the possibility of altering adhesive properties, increasing the mechanical and physical properties of wood through reinforcement, and reducing FEs.

## Overview of Emerging Technologies and Reaction Mechanisms

### Binderless WBCPs

Binderless panels contain no adhesive and are produced by activating the chemical components of the wood panel during steam or heat treatment [96]. When heat and pressure are applied to lignocellulosic material, the constituent components of wood cell walls—cellulose, hemicelluloses, and lignin—degrade. This degradation yields monomers containing free radicals derived from hemicelluloses, amorphous cellulose, and lignin with additional reactive sites. These reactive sites react again, which promotes self-bonding in binderless panels. Moreover, the degradation of lignin and hemicelluloses produces acetone, organic acids, and furfural acids, which can undergo polymerization and serve as effective binding agents [97]. To produce a sufficient bonding area, particularly in the absence of a binder, wood polymers must be plasticized above their glass transition temperature. Consequently, when lignocellulosic material is subjected to high temperatures, its components undergo reactions that lead to the release of acidic compounds from hemicelluloses. These compounds act as catalysts for cellulose and hemicellulose degradation and then recondense to form a natural resin or bond with lignin. The plasticized lignin envelops the cellulose and hemicelluloses, and solidifies as the temperature decreases towards the end of the process. This process significantly influences the mechanical properties of the final material. Due to the wide variety of components and characteristics it can have, lignocellulosic material is well-suited for producing binderless panels [98]. Various parameters, including temperature, pressure, time, and particle size, affect the self-bonding mechanism. In self-bonding processes, the main reactions often involve the activation of chemical components within the panel constituents, particularly through hemicellulose hydrolysis and lignin softening during hot pressing. Additionally, investigating chemical and enzymatic pretreatments on raw materials presents innovative strategies for improving binderless particleboard production. Chemical pretreatments such as alkaline, acidic, and oxidation agents have been used to activate fiber surfaces [99]. In contrast, enzymatic treatments typically involve milder reaction conditions, produce fewer by-products, and are more environmentally friendly compared to chemical alternatives.

–Mechanical Pretreatments

Mechanical pretreatment increases the total accessible surface area and thus improves the accessibility of constituents and leads to better bonding strength. Refining wood particles through the grinding process leads to a reduction in crystallinity of cellulose, particle size, and polymerization degree. However, achieving fine grinding necessitates a considerable energy input. The size and shape of the particles can significantly affect the properties of binderless panels. Particleboard with particles measuring 0.25 to 1 mm has been shown to have the best mechanical results [100]. To enhance the dimensional stability and adhesion of binderless fiberboard, steam explosion is employed to increase the contact area through defibrillation, thus resulting in a rough fiber surface. Moreover, this process releases lignin from the cell wall, which acts to prevent the water absorption of hydrophilic polymers like hemicelluloses and cellulose [101].

–Chemical Pretreatments

Chemical pretreatments can be acidic, neutral, or alkaline. Commonly employed acids include sulfuric acid, hydrochloric acid, and acetic acid, which are utilized for lignocellulosic component hydrolysis. Acid pretreatment effectively breaks the van der Waals forces, hydrogen bonds, and covalent bonds, and leads to the solubilization of hemicelluloses, the partial breakdown of lignin, and an increase in cellulose crystallinity. Therefore, alkaline and acid concentrations and treatment times must be carefully controlled to avoid the excessive degradation of the main components of the wood. Moreover, under acidic catalysis, hemicelluloses can generate furfural and 5-HMF, with furan monomers produced during high-temperature wood heating. Lewis acids, like aluminum chloride hexahydrate (AlCl_3_·6H_2_O), have been shown to promote furfural and 5-HMF generation, which has the potential to promote wood fiber self-bonding. Alkaline pretreatment involves alkaline reagents, such as sodium hydroxide and potassium hydroxide in aqueous solutions, being added to biomass. This pretreatment results in increased internal surface area through cell wall swelling, a reduction in the degree of polymerization and crystallinity, the breaking of links between lignin and other polymers, and the breakdown of lignin. NaOH pretreatment facilitates the saponification of intermolecular ester bonds [101].

–Biological Pretreatments

Enzymatic pretreatment can activate the surface by breaking down molecules and generating additional reactive sites, such as radicals, for self-bonding, which improves binderless fiberboard performance. Phenol-oxidizing enzymes, such as laccase and peroxidases, are produced by various organisms, including fungi—particularly white rot—as well as certain plants, bacteria, and animals. These enzymes, expressed notably by white-rot fungi, can degrade cellulose, hemicelluloses, and lignin. Both laccase and peroxidases facilitate the one-electron oxidation of phenolic groups into phenoxy radicals; however, their mechanisms differ. Laccase catalyzes the oxidation of phenolic substrates using dioxygen (O_2_), while peroxidases like lignin peroxidase and manganese peroxidase rely on hydrogen peroxide as a substrate [102]. Since laccase is an oxidoreductase agent, it removes the lignin from cellulosic material and promotes lignin polymerization via free radical reactions. Laccase penetration into fibers during treatment is limited and results in primarily surface lignin oxidization. Consequently, the fiber surface generates free radicals, which serve as reactive sites for subsequent crosslinking reactions during fiberboard production [103]. The laccase oxidation mechanism is described as follows (1):

1 $$4Phe-OH+{O}_{2}\to 4Phe-{O}^{\bullet }+2{H}_{2}O$$

Another approach is to produce what are called mycelium-based bio-composites (MBCs) by incorporating the mycelia of filamentous fungi in a substrate and nutrient blend in a predeveloped mold. The mycelia act as a binder by efficiently weaving an entangled network that binds the substrate particles together into a cohesive aggregation. In industrial settings and research laboratories, oven drying is frequently utilized to inhibit fungal growth and produce the final product [104]. The fungi most commonly used to pretreat lignocellulosic bio-composites are white-rot fungi such as *Trametes* sp., *Colorius* sp., *Pleurotus* sp., *Pycnoporus* sp., and *Ganoderma* sp. It is preferable to use a selective white-rot fungus that will degrade lignin and hemicelluloses, while leaving cellulose mostly unaltered, as it predominantly contributes to the material's strength. The steps involved in MBC production include materials selection (fungal strain and feedstock), substrate sterilization, inoculation, molding, incubation, heat treatment (from 130 °C to 200 °C), and surface finishing [105].

Enzymatic and fungal pretreatments are categorized as wet processes, although the substrate can undergo pre-drying before the pressing stage. Enzymes are preferred for pretreating higher-density fiberboard, while fungi are commonly employed for lower-density particleboard. However, the results prove challenging due to the considerable influence that various parameters such as time, pH, temperature, and relative humidity have on the efficacy of fungal and enzymatic pretreatments [104].

### Esterification and Imidization Reactions

Ando et al. attempted to understand the reactions between citric acid and wood. They proposed that esterification occurs between citric acid and various wood components. Their findings revealed that, under the wood molding conditions utilized, citric acid partially esterifies the primary hydroxyl groups of polysaccharides such as cellulose and glucomannan, as well as certain lignin substructures like β-O-4 and β-5, along with a secondary hydroxyl group at the 2-position of xylan [106]. The formation of covalent bond ester linkages contributes to the excellent dimensional stability and water resistance exhibited by wood moldings bonded with citric acid-type adhesive. Scharf et al. developed a novel formaldehyde-free adhesive system for particleboard based on imidazole in combination with citric acid and sorbitol. Nonetheless, the mechanism underlying the interaction between imidazole and wood / citric acid remains uncertain [107]. Chen et al. used an olefin-maleamic acid (OMA) resin to develop a formaldehyde-free adhesive for WBCPs. Their research demonstrated that the amic acid groups in the OMA resin can undergo two distinct reactions when subjected to hot pressing. The first is an anhydration reaction, which leads to the formation of anhydride groups, and the second is an imidization reaction, which results in imide group formation. The imidization reaction tends to predominate and imparts excellent water-resistance properties to WBCPs. Meanwhile, the anhydride groups produced by the anhydration reaction can further interact with the hydroxyl groups of wood through esterification to facilitate the crosslinking of wood material [108]. Glycerol and maleic anhydride, derived from biomass, are utilized as the primary constituents for the esterification process. Ring-opening grafting maleic anhydride onto the three hydroxyl groups of glycerol results in polyacids. Subsequently, the synthesis of a hyper-branched polyester (HBPE) is achieved by adding more glycerol, which leads to the condensation of glycerol's hydroxyl groups with the carboxyl groups of polyacids. HBPE resin has been tested as an adhesive for wood, and the best bonding performance was achieved when the reaction time between the polyacids and glycerol was two hours. The reaction temperature was 140 °C [109]. Furthermore, the catalytic oxidation of glycerol yields various compounds like glyceraldehyde and dihydroxyacetone.

Finally, Table 2 presents a comprehensive overview of various bio-based technologies and adhesives considered in this work along with their respective applications in WBCPs.

**Table 2:** Summary of the various bio-based technologies and wood adhesives cited in this review, and their applications in WBCPs

Technology	Adhesive	Method	Application	Reference
Bio-based materials	Tannin-based	Tannins with hardeners, proteins, sucrose, starch, lignin	Interior panels, particleboard, plywood	[65–71, 73]
	Starch-based	Modification by chemical processes, grafting, degradation	Interior panels, particleboard, plywood, MDF	[54, 56, 57, 70, 110]
	Chitosan-based	Modification by chemical processes or reaction with acids and aldehydes	Plywood, MDF	[58, 59]
	Sucrose-based	Esterification of sucrose with acids, sucrose degradation, oxidation processes, and reaction with hardeners	Plywood	[61, 63]
	Lignin-based	Modification by demethylation, hydroxymethylation, phenolation, and oxidation	Interior and exterior particleboard panels, plywood	[34, 38, 39, 42, 43]
	Protein-based	Modification by thermal, chemical, enzymatic, and mechanical methods	Particleboard, MDF, plywood	[28, 30, 48, 49, 73]
Binderless panels	None	Mechanical treatment, chemical treatment, biological treatment	Interior panels, particleboard, MDF	[96–101, 103, 105]
Reactive systems	Esterification	Formation of ester linkages	Interior panels, particleboard, MDF, plywood	[106, 108, 109]
	Imidization	Formation of imine linkages		

### Bio-based vs. Synthetic Adhesives: Life Cycle Analysis and Eco-friendliness

The life cycle assessment (LCA) methodology has been proven to be the most appropriate way to assess the environmental impacts of products. LCA can consider the entire life cycle of a product, i.e., from the sourcing of raw materials through processing, manufacturing, and marketing until the end of its life. Several studies have highlighted that the significant environmental footprint of WBCPs primarily stems from the utilization and processing of wood resources, as well as the FE released by synthetic adhesives [103]. Several LCA comparison studies have shown that bio-based adhesives sourced from diverse origins demonstrate better environmental performance compared to petrochemical-based adhesives in WBCP manufacturing. For instance, Arias et al. conducted comprehensive LCAs of lignin (kraft and organosolv), soy, and tannin-based adhesives, and compared their results with those of their petrochemical-based counterparts [111]. In lignin-based adhesives, the glyoxylated lignin functionalization stage emerges as the primary contributor to their significant environmental impact, particularly due to substantial energy demands and unregulated glyoxal emissions into the air. Existing examinations of tannin-based adhesives have focused on enhancing on-site emissions and optimizing the chemical dosage. Two main concerns have been highlighted: the need to mitigate terrestrial toxicity resulting from uncontrolled glyoxal emissions, and the need to minimize the glyoxal dosage while manufacturing tannin-based bio-adhesives. Finally, soy-based adhesives can be considered an environmentally promising alternative to fossil-based resins, as their overall impact is approximately 25% less than that of PF adhesives and nearly equivalent to that of UF adhesives. However, Moretti et al. compared three previous LCA of adhesives used in fiberboard production and found there were variations between the overall performance evaluations of the bio-based adhesives and those of their petrochemical counterparts [112]. The outcomes depended on the type of lignin used and the underlying assumptions made. In one scenario, the environmental performance of lignin-based adhesives was significantly worse than that of conventional formaldehyde-based adhesives. In another instance, an LCA comparison suggested that WBCPs produced with lignosulfonate-based adhesives were environmentally superior to those produced using UF adhesive. Similarly, kraft lignin-based adhesives were deemed more environmentally friendly than UF adhesives [112]. Finally, Eisen et al. highlighted that the environmental efficiency of industrial bio-based adhesives remains a subject of considerable debate, primarily relying on assumptions regarding theoretical and unverified products. A significant obstacle to the widespread adoption of innovative bio-based value products is the fact that many conversion technologies are still in experimental or pilot phases [113]. Ultimately, Heinrich emphasized two factors that are crucial for any alternative adhesive. Firstly, its pricing will significantly influence its market adoption, and several studies have indicated that bio-based adhesives can be cheaper than their petrochemical-based counterparts. Secondly, the renewable products' success hinges on its ability to function as a drop-in replacement for conventional practices without requiring any changes to manufacturing facilities or equipment [15].

## Conclusions and Perspectives

This review highlights the progress that has been made in utilizing coproducts as primary materials to produce bio-based adhesives for WBCPs. Significant breakthroughs have been achieved by exploring various resources derived from plant and animal origins, and resulted in bio-based adhesives being developed with minimal or no formaldehyde content. These resources span a wide variety of materials, including lignocellulosic compounds, tannins, proteins, and saccharides. By redirecting these resources away from conventional disposal methods like landfills or incineration, industries stand to mitigate the substantial cost burden associated with disposal processes while fostering the development of high-value-added materials like adhesives. Despite lingering challenges, such as the limited water resistance and bonding strength of bio-based adhesives, ongoing efforts in terms of material modification and the exploration of diverse hardeners and crosslinkers offer promising avenues for improvement and enabling bio-based adhesives to compare favorably with their synthetic counterparts. Innovative bio-scavengers are being actively developed in the quest to reduce FEs, as is highlighted in this review.

Additionally, emerging technologies such as binderless panels, which circumvent the need for any adhesive by activating the chemical components of the panel constituents to adhere the wood particles through mechanical, chemical, hydrothermal, or biological treatments, are gaining prominence in scientific research. Furthermore, growing emphasis is being put on modifying wood particles to catalyze esterification reactions between acids and various wood components and open up new pathways for adhesive innovation.

In conclusion, despite methodological variations yielding different outcomes, LCA comparison studies consistently underscore the potential of bio-based adhesives to supplant their traditional counterparts and thereby reduce the negative impacts of WBCPs on human health and the environment. Realizing this potential hinges on advancements in extraction techniques, the adoption of sustainable modification processes, and the implementation of energy-saving measures throughout the life cycle of bio-based adhesives for the wood panel industry.

## Data Availability

No datasets were generated or analysed during the current study.
